# Effects of transcranial direct magnetic stimulation in patients with primary progressive aphasia: a systematic review

**DOI:** 10.1002/alz70858_107604

**Published:** 2025-12-25

**Authors:** Vitório Serafim, Gabrielle Guindani Maia, Leonardo Bedatti Koehler, Ana Júlia Rodrigues Ribeiro, Morghana Machado da Rosa, Vitor Ritt Xavier, Ana Carolina Sgarioni Viapiana, Rodrigo de Cezaro, Cavaler Juvêncio, Maria Fernanda Peruci Felippe, Gabriela Bezerra Sorato

**Affiliations:** ^1^ Fundação Universidade Federal de Ciências da Saúde de Porto Alegre, Porto Alegre, Rio Grande do Sul, Brazil; ^2^ Universidade Federal do Rio Grande do Sul, Porto Alegre, Rio Grande do Sul, Brazil

## Abstract

**Background:**

Transcranial direct current stimulation (tDCS) is a non‐invasive intervention for brain dysfunction which can be used as treatment in patients with primary progressive aphasia (PPA), a neurodegenerative disorder divided into three variants: logopenic (lvPPA), semantic (svPPA) and nonfluent/agrammatic (nfvPPA). This systematic review aims to analyze the effects of tDCS in PPA patients.

**Method:**

An active search was performed in PubMed, Embase and Cochrane databases, using the keywords ‘transcranial magnetic stimulation’, ‘primary progressive aphasia’ and their equivalents. Two reviewers independently evaluated the titles and abstracts of studies published before January 2025. Duplicate results were eliminated and only complete randomized controlled trials in English that had the evaluation of the use of tDCS on PPA patients as their objective were included.

**Result:**

85 articles were identified, with 5 meeting inclusion criteria totaling 119 patients. The studies showed no significant differences in the effects of tDCS were found between different PPA variants. When applied to the dorsolateral prefrontal cortex with anodal tDCS, 2 studies showed a significant effect in the naming subtest, with improvement over time (*p* = 0.018) and naming accuracy after 12 and 24 weeks (*p* = 0.049) (*p* = 0.030). In other brain regions applied with high‐definition tDCS, no significant difference was observed in the overall aphasia score analysed (*p* = 0.14). Although, the naming subtests revealed a positive effect of tDCS (*p* = 0.02). While the results were positive for men, in women tDCS significantly reduced functional connectivity (FC) in the Language Network (*p* < 0.001), suggesting a possible interference in linguistic processing. Furthermore, aging was associated with a reduction in the FC of this same network (*p* = 0.009). However, energy and speech production effects were seen momentarily, they did not persist in the long term. 1 study demonstrated the general pattern of outcomes showing improvement immediately following interventions and decaying over time.

**Conclusion:**

tDCS demonstrated efficacy in improving naming abilities, but did not show sustained long‐term effects for energy and speech production. Furthermore, the findings indicate that FC responds differently to electrical stimulation, varying according to sex and age.

